# Functional Advantages of *Porphyromonas gingivalis* Vesicles

**DOI:** 10.1371/journal.pone.0123448

**Published:** 2015-04-21

**Authors:** Meng-Hsuan Ho, Chin-Ho Chen, J. Shawn Goodwin, Bing-Yan Wang, Hua Xie

**Affiliations:** 1 School of Dentistry, Meharry Medical College, Nashville, Tennessee, United States of America; 2 Department of Surgery, Duke University Medical Center, Durham, North Carolina, United States of America; 3 Department of Biochemistry and Cancer Biology, Meharry Medical College, Nashville, Tennessee, United States of America; 4 Department of Periodontics, School of Dentistry, University of Texas, Health Science Center at Houston, Houston, Texas, United States of America; University of Oklahoma Health Sciences Center, UNITED STATES

## Abstract

*Porphyromonas gingivalis* is a keystone pathogen of periodontitis. Outer membrane vesicles (OMVs) have been considered as both offense and defense components of this bacterium. Previous studies indicated that like their originating cells, *P*. *gingivalis* vesicles, are able to invade oral epithelial cells and gingival fibroblasts, in order to promote aggregation of some specific oral bacteria and to induce host immune responses. In the present study, we investigated the invasive efficiency of *P*. *gingivalis* OMVs and compared results with that of the originating cells. Results revealed that 70–90% of human primary oral epithelial cells, gingival fibroblasts, and human umbilical vein endothelial cells carried vesicles from *P*. *gingivalis* 33277 after being exposed to the vesicles for 1 h, while 20–50% of the host cells had internalized *P*. *gingivalis* cells. We also detected vesicle-associated DNA and RNA and a vesicle-mediated horizontal gene transfer in *P*. *gingivalis* strains, which represents a novel mechanism for gene transfer between *P*. *gingivalis* strains. Moreover, purified vesicles of *P*. *gingivalis* appear to have a negative impact on biofilm formation and the maintenance of *Streptococcus gordonii*. Our results suggest that vesicles are likely the best offence weapon of *P*. *gingivalis* for bacterial survival in the oral cavity and for induction of periodontitis.

## Introduction

Bacterial vesicles are produced generally by Gram-negative bacteria through the blebbing and pinching-off of the bacterial outer membrane [1]. Bacterial vesicles have been found in culture media, samples of dental plaque and even the open ocean [2–5]. Using nanoparticle tracking analysis, Biller *et al*. recently demonstrated that vesicles in the culture medium of *Porchlorococcus* were as abundant as the number of cells in early growth phase, while they were 10 times as numerous as the number of cells found in the exponential and stationary phases [2]. As they carry many of the virulence factors found in bacterial outer membranes and the periplasm, vesicles are known to be an efficient vehicle involved in the pathogenicity of Gram-negative bacteria. Studies have shown that vesicles are involved in adherence, biofilm formation, invasion, host cell damage, and modulation of host immune responses [6]. They have distinct functional advantages over whole bacterial cells. For example, vesicles that are enriched in bacterial virulence factors and signal molecules are protected from dilution and proteolytic degradation and are able to travel to distant targets. Thus *Pseudomonas aeruginosa* packages 2-heptyl-3-hydroxy-4-quinolone into vesicles that transport this molecule within a population of cells to communicate and coordinate social activities of the bacteria [7].

*P*. *gingivalis*, in particular, is a gram-negative bacterium associated with chronic periodontitis. Vesiculation in *P*. *gingivalis* was first reported in the 1980s [8]. Earlier studies demonstrated that the vesicles serve as a vehicle for toxins, proteolytic enzymes, and adhesins, based on observations that vesicles were able to: 1) degrade collagen, Azocoll, and N-alpha-benzoyl-DL-arginine p-nitroanilide, and 2) promote bacterial adherence between two non-coaggregating bacterial species such as *Capnocytophaga ochracea* and *Eubacterium saburreum* [3]. Previous studies revealed that *P*. *gingivalis* vesicles were also able to enhance the attachment and invasion of *Tannerella forsythia* to epithelial cells. They were also able to mediate coaggregation between *Treponema denticola* and *Lachnoanaerobaculum saburreum* and between mycelium-type *Candida albicans* and *Staphylococcus aureus* [9–11], processes which did not occur in the absence of *P*. *gingivalis* vesicles or in the presence of heat-treated vesicles. Recent studies using proteomic tools have validated that *P*. *gingivalis* vesicles may act as intermediaries that carry a wide variety of virulence factors provided by their parent cells. Most virulence factors displayed on the outer membrane of *P*. *gingivalis* were found in the vesicles, including FimA, Mfa1, HagA, and gingipains [12,13]. More interestingly, some well-known virulence factors were enriched in vesicles when compared to levels found in *P*. *gingivalis* cells. We recently reported an approximately 3–5 fold increase in gingipain levels in vesicles compared to the levels observed in surface extracts of the originating cells [14]. Among the functional advantages of *P*. *gingivalis* vesicles, was the finding that they were able to induce immune responses. Using a mouse model, Nakao *et al*. demonstrated that vesicles effectively elicited *P*. *gingivalis*-specific serum IgG and IgA as well as salivary IgA five weeks after an initial intranasal immunization, whereas whole cells did not [15]. It was suggested that the increased antigenicity found in the vesicles might result from the more concentrated immune-dominant determinants on the vesicles compared to *P*. *gingivalis’* cell surfaces.

Although *P*. *gingivalis* vesicles have been intensively studied recently, the virulence features of the vesicles with respect to periodontitis, especially when compared to *P*. *gingivalis* cells, are not completely understood. In order to further define the functional advantages conferred by *P*. *gingivalis* vesicles, we determined the invasive efficiencies of both *P*. *gingivalis* cells and vesicles and role of *P*. *gingivalis* vesicles in biofilm formation. Moreover we now show that a previously unrecognized property of *P*. *gingivalis* vesicles, that of horizontal gene transfer. These findings may represent an opportunity to utilize characteristics of *P*. *gingivalis* vesicles to develop strategies to reduce bacterial virulence.

## Materials and Methods

### Bacterial strains and vesicle preparation

*P*. *gingivalis* strains are listed in Table 1, They were grown from frozen stocks in TSB (trypticase soy broth) or on TSB blood agar plates supplemented with yeast extract (1mg/ml), hemin (5 μg /ml) and menadione (1μg/mL), and incubated at 37°C in an anaerobic chamber (85% N2, 10% H2, 5% CO2). Streptococcus strains were grown in Trypticase Peptone Broth (TPB) supplemented with 0.5% glucose at 37°C under aerobic conditions. *P*. *gingivalis* vesicles were prepared as previously described [16]. Briefly, *P*. *gingivalis* was grown to the late exponential phase and growth media were collected by centrifugation at 10,000 × *g* for 15 min at 4°C and filtered through a 0.22-μm-pore-size filters (CellTreat) to remove residual bacteria. Vesicles were collected by ultracentrifugation at 126,000 x *g* for 2 h at 4°C and resuspended in phosphate-buffered saline (PBS) containing 10% glycerol. Since quantifying vesicles by their protein or lipid content in weight represents the most common way to normalize data [1], for these studies proteins were extracted from vesicles using a BugBuster Protein Extraction Reagent (Novagen) and protein concentrations were determined with a Bio-Rad Protein Assay Kit (Bio-Rad).

**Table 1:** Bacterial strains used in this study.

Strains	Relevant characteristics^*a*^	Source or reference
*P*. *gingivalis* 33277	Type strain from ATCC, *fimA* type I	Lab collection
*P*. *gingivalis* 49417	Type strain from ATCC, *fimA* type III	Lab collection
*P*. *gingivalis* W83	Type strain from ATCC, *fimA* type IV	Lab collection
*P*. *gingivalis* MFAE	Derivative of *P*. *gingivalis* 33277 containing an insertional mutation in *mfa1*, Em^r^	[51]
*P*. *gingivalis* FAE	Derivative of *P*. *gingivalis* 33277 containing an insertional mutation in *fimA*, Em^r^	[22]
*P*. *gingivalis* KDP128	Derivative of *P*. *gingivalis* 33277 containing insertional mutations in *rgpA/rgpB/kgp*, Em^r^/Tet^r^	[26]
49417_Δ*fimA* mutant	Derivative of *P*. *gingivalis* 49417 containing an insertional mutation in *fimA*, Em^r^	[22]
*S*. *gordonii* DL1	Wild-type strain	Lab collection

^*a*^ Em^r^, resistance to erythromycin, and Tet^r^, resistance to tetracycline.

### Treatment of host cells with *P*. *gingivalis* vesicles.

Human gingival fibroblasts (HGF) and human oral keratinocytes (HOK) were purchased from ScienCell Research Laboratories and human umbilical vein endothelial cells (HUVEC) from ATCC. These host cells were cultured in specific media, respectively according to the manufacturer’s instructions. Prior to treatment, HGFs, HOKs, and HUVECs (2 × 10^4^) were seeded and grown overnight in poly-L-lysine coated 35mm glass bottom dishes (MatTek Corporation, Ashland, MA), and then exposed to *P*. *gingivalis* 33277 (2 × 10^6^) or its vesicles (100 ng) for 0 or 1 h. After removal of unbound vesicles, HGFs were cultured for another 3 or 24 h, at 37°C, 5% CO2, using fresh media. The cytotoxicity of vesicle treatment was evaluated with a Pierce LDH Cytotoxicity Assay Kit (Thermo Scientific). There was no cytotoxicity detected under our experimental conditions.

### Confocal microscopy

HOKs and HGFs were fixed with 3.8% formaldehyde in a sodium phosphate buffer at room temperature for 10 min after treatment, permeabilized with 0.1% Triton X-100 for 10 min, and blocked with 5% bovine serum albumin in PBS for 1 h. HOKs and HGFs were then immunostained with pan-specific antibodies of *P*. *gingivalis* 33277 and followed by goat anti-rabbit IgG conjugated to Alex Fluor 546 (Invitrogen). Nuclei were stained with 4′, 6-diamino-2-phenylindole (DAPI, 1 μg/mL; Invitrogen). Confocal images were acquired using a Nikon A1R confocal microscope.

### Vesicle-associated DNA and RNA purification

To eliminate any surface-associated DNA and free DNA in the suspension, vesicles (5 μg) purified from *P*. *gingivalis* 33277 cultures were resuspended in PBS and treated with or without 2U of DNase I (Invitrogen) in a 30 μl final reaction volume, and incubated at 37°C for 30 min according to the manufacturer’s instructions. Any DNase I was then inactivated at 75°C for 15 min, and the protected DNA inside of vesicles was released by treatment of vesicles at 100°C for 10 min. The samples were then centrifuged at 16,000 × *g* for 5 min. The supernatants containing the DNA were collected for PCR analysis. For vesicle-associated RNA purification, vesicle samples (25 μg) were treated with 5 μl of GES lysis buffer (50 mM guanidinium thiocyanate, 1 mM EDTA, and 0.005% (w/v) sarkosyl, final concentration) in a 100 μL final reaction volume [2]. RNA was purified from the reaction mixture using an Aurum Total RNA Mini Kit (Bio-Rad) following the manufacturer’s protocol.

### PCR and real-time PCR analyses

The extracted DNAs were amplified by PCR in a 50 μL final reaction volume containing 1 μL of the supernatant of heat treated vesicles, the primers listed in S1 Table, and 0.2 μL of Platinum Taq DNA polymerase (Invitrogen). The amplifications were performed in a thermal cycler (Bio-Rad) at 94°C for 45 s, 56°C for 30 s, and 72°C for 30 s for a total 30 cycles, followed by 10 min of elongation at 72°C. PCR products were separated by agarose gel electrophoresis, stained with ethidium bromide, and visualized by UV transillumination with an EpiChemi III Darkroom (UVP). To detect RNA in *P*. *gingivalis* vesicles, RNAs were reverse-transcribed using iScript reverse Transcription Supermix (Bio-Rad), and real-time RT-PCR was performed by using an SsoAdvanced SYBR Green Supermix on a C1000 Touch Thermal Cycler (Bio-Rad) according to the manufacturer's instructions. RNA levels in vesicle samples were determined relative to the non-reverse transcribed calibrator sample by using the comparative cycle threshold (*ΔC*_*T*_) method. *ΔC*_*T*_ was calculated by subtracting the average *C*_*T*_ value of the test sample from the average *C*_*T*_ value of the calibrator sample, and was then used to calculate the ratio between the two by assuming a 100% amplification efficiency.

### Horizontal DNA transfer with vesicles

Gene transfer between *P*. *gingivalis* cells and vesicles was examined using vesicles isolated from a mutant strain generated from *P*. *gingivalis* 49417 by introducing a 2.1-kb *ermF-ermAM* cassette into the *fimA* gene. Since the *erm* resistant gene was detectable in these vesicles, we used these vesicles as donors in a DNA transfer assay. In brief, *P*. *gingivalis* 33277 was anaerobically cultured in TSB at 37° for 6 h to reach an optical density at 600 nm (OD_600_) of 0.5. Cells (10^8^) were pelleted, resuspended, and mixed with the vesicles (2.5 μg) in 1 ml of fresh TSB. The suspension was incubated for 24 h, and recipients were selected by plating the bacterial suspension on TSB blood plates supplemented with erythromycin (5 μg/mL). After culturing for 5–7 days, the colonies were counted and collected for future PCR analysis. To confirm that the *erm* resistant gene was indeed incorporated into the *fimA* gene, *P*. *gingivalis* chromosomal DNA was isolated from the recipients using an Easy-DNA Purification Kit (Invitrogen). PCR was performed using DNA isolated from the recipients as a template, while the primers corresponded to sequences of the *fimA* gene.

### Biofilm formation and dispersal of *Streptococcus gordonii*

Attachment of *S*. *gordonii* DL1 to human saliva coated surfaces was quantified by the method of O’Toole and Kolter [17]. Human whole saliva was collected from periodontally healthy donors. The saliva collection protocol was approved by the IRB of Meharry Medical College and all participants gave informed written consent. After centrifugation and filter sterilization (pore size, 0.22 μm) to remove cellular debris, saliva was incubated in a 96-well polystyrene plate (Corning Incorporated) for 1 h. The plate was then washed with phosphate-buffered saline (PBS, 100 mM NaH2PO4, 150 mM NaCl). *S*. *gordonii* DL1 was grown in TPB to mid log phase (OD600 = 1.0) and collected by centrifugation. The bacterial cells were resuspended in fresh TPB and added to each well (5×10^7^/well) of a 96-well plate that was pre-coated with human whole saliva with or without *P*. *gingivalis* vesicles, and then incubated at 37°C in an anaerobic chamber for 24 h. To determine the role of *P*. *gingivalis* vesicles in biofilm dispersal of *S*. *gordonii*, the vesicles were added to the wells including an *S*. *gordonii* biofilm in ¼ TPB (1 vol phosphate buffered saline TBP and 3 vol PBS) and incubated for another 24 h before biofilm quantitation measures. The bacterial biofilms were stained with 1% crystal violet for 15 min. After washing with PBS three times, the biofilms were destained with 200μL of 95% ethanol for 30 sec; 125μL of absolute ethanol from each well was transferred to a fresh well for reading at 570 nm in a Benchmark Plus Microplate Spectrophotometer (Bio-Rad).

### Statistical analyses

A student’s *t*-test was used to determine statistical significance of the differences in the invasive activities of *P*. *gingivalis* cells and vesiclesm and on the effect of the vesicles on *S*. *gordonii* biofilms. A *P*<0.05 was considered significant. Values are shown ± SD unless stated otherwise.

## Results

### Comparison of the invasive efficiency between *P*. *gingivalis* vesicles and their originating cells

**Fig 1:**
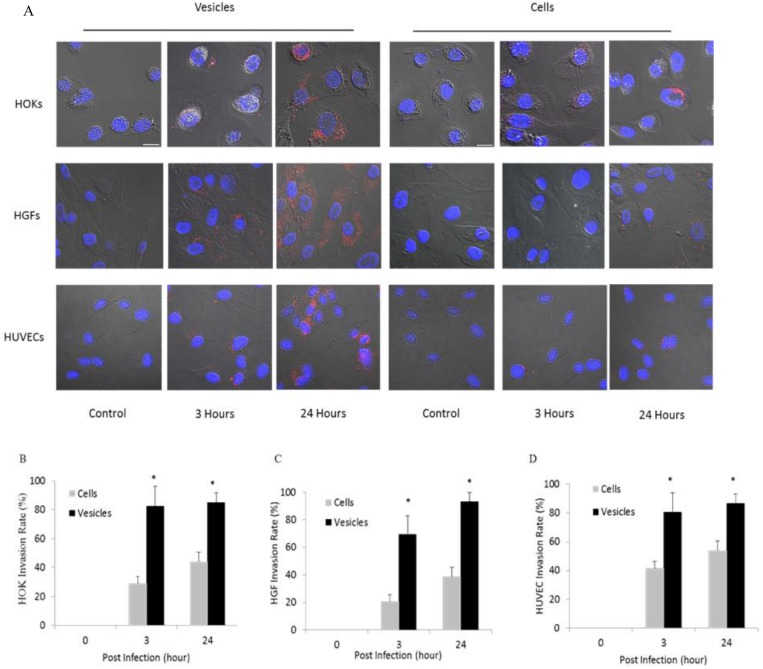
Invasive activity of *P*. *gingivalis* vesicles into HOKs, HGFs, and HUVECs. Infected cells presented by differential interference contrast (DIC) images. *P*. *gingivalis* cells and vesicles were stained with primary anti-33277 serum and a secondary antibody conjugated with Alex Fluor 546 (red); nuclei were stained with DAPI (blue) (A). Scale bars, 20 μm. The numbers of HOKs (B), HGFs (C), and HUVECs (D) with intercellular *P*. *gingivalis* and vesicles were determined by counting ten random areas. Each bar represents the percentage of HOKs, HGFs, and HUVECs with intercellular bacteria or vesicles. The SEs are indicated (*n* = 3). An asterisk indicates the statistical significance of invasive rates between *P*. *gingivalis* 33277 cells and their derived vesicles (*P* <0.05; t test).

Our recent study showed that *P*. *gingivalis* OMVs were able to efficiently invade HOKs [14]. We further compared the invasive activities of *P*. *gingivalis* cells and vesicles. To expose HOKs, HGFs, and HUVECs to *P*. *gingivalis* 33277 cells or their vesicles, host cells (2 × 10^4^) were cultured with *P*. *gingivalis* cells (2 × 10^6^) or vesicles (100 ng, equivalent to vesicles released by 2 × 10^4^ of *P*. *gingivalis* in late log-phase) [18] for 1 h. After removal of the unbound vesicles, cells were cultured for another 3 or 24 h with fresh media. The invasive efficiency of *P*. *gingivalis* and its derived vesicles were examined under a confocal microscope. After exposure to *P*. *gingivalis* cells or vesicles, respectively, both the bacterial cells and the vesicles were found in HOKs, HGFs, and HUVECs but with significantly different efficiencies (Fig 1A). By counting 10 random areas (1.34 × 1.34 mm), we found that 82 ± 5.4, 69 ± 6.5, and 80 ± 13.4% of the HOKs, HGFs, and HUVECs engulfed vesicles 3 h post exposure, respectively, and up to 85 ± 7.1, 93 ± 2.9, and 87 ± 6.9% of the HOKs, HGFs, and HUVECs contained the vesicles after a 24 h exposure (Fig 1B, 1C, and 1D). Far more vesicles were internalized in individual HOKs or HGFs at 24 h than at 3 h, as shown by the greater intensity of red fluorescence. We observed a significantly lower invasive efficacy of *P*. *gingivalis* cells compared to vesicles. Two to three fold less the host cells carried intercellular bacteria 3 h post exposure compared to the number of the cells with intercellular vesicles. Moreover, none of the host cells were found with engulfed bacteria or vesicles when the bacteria or vesicles were immediately removed after exposure (0 h values). These data provide strong evidence that *P*. *gingivalis* vesicles possess a higher invasive activity than their parent cells.

### Detection of DNAs and RNAs in *P*. *gingivalis* vesicles

**Fig 2:**
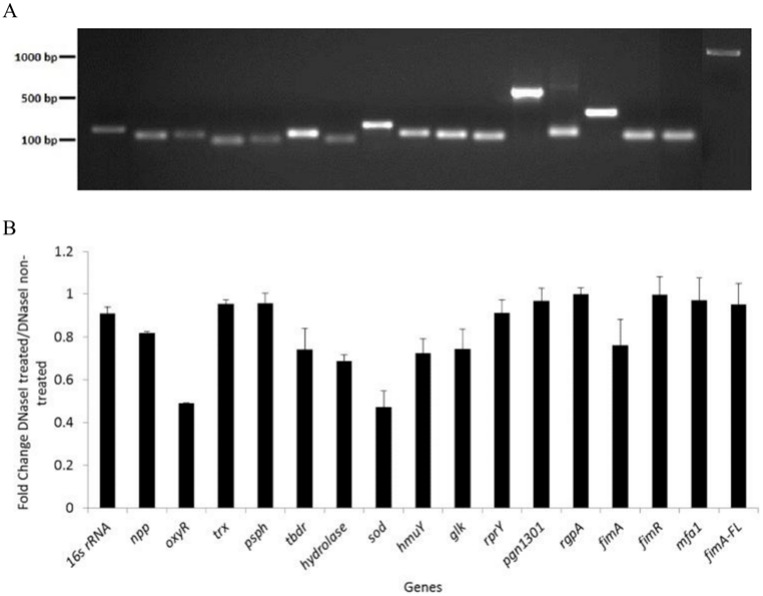
Detection of DNA fragments in *P*. *gingivalis* vesicles. (A) PCR amplification was performed with DNA released from *P*. *gingivalis* 33277 vesicles. PCR products were visualized on agarose gels. (B) Semiquantitation of PCR products was conducted with ImageJ software. Each bar represents the intensity of the PCR product band from amplification of DNA in vesicles treated with DNase I relative to that in vesicles without DNase I treatment. Means and SDs are indicated (*n* = 3).

Nucleic acids have been found as components in outer membrane vesicles of some bacteria [2,19]. To test if *P*. *gingivalis* vesicles carry any DNA fragments, we first pre-treated purified vesicles with or without DNase, and any residual protected DNA was released from vesicles by heat treatment. The DNA was amplified using PCR with16 primer pairs specifically corresponding to *P*. *gingivalis* genes, and included some well-known virulence genes. As shown in Fig 2A, all DNA fragments tested were amplified, although at different efficiencies. DNA fragments of genes encoding the major subunit of long fimbriae (*fimA*) and superoxide dismutase (*sod*) were detected at relatively higher levels, suggesting there may be a preferential internal packaging of DNAs by *P*. *gingivalis* vesicles. To further examine if some of the DNA fragments were large enough to encode a gene, we amplified and detected a 1.2 kilobase of the *fimA* gene including its promoter and terminator regions (*fimA*-FL, Fig 2A), indicating that some DNA in vesicles is large enough to encode a virulence factor. When comparing levels of PCR products using DNA templates from vesicles with or without DNase pre-treatment, we found that level of DNA amplification with certain specific primer pairs was decreased after DNase treatment (Fig 2B). This suggests that cytoplasmic DNAs may not only be packaged into vesicles, but also bind to the surfaces of the vesicles. The latter would likely be degraded when it voyages through oral microbial biofilms and periodontal tissues.

**Fig 3:**
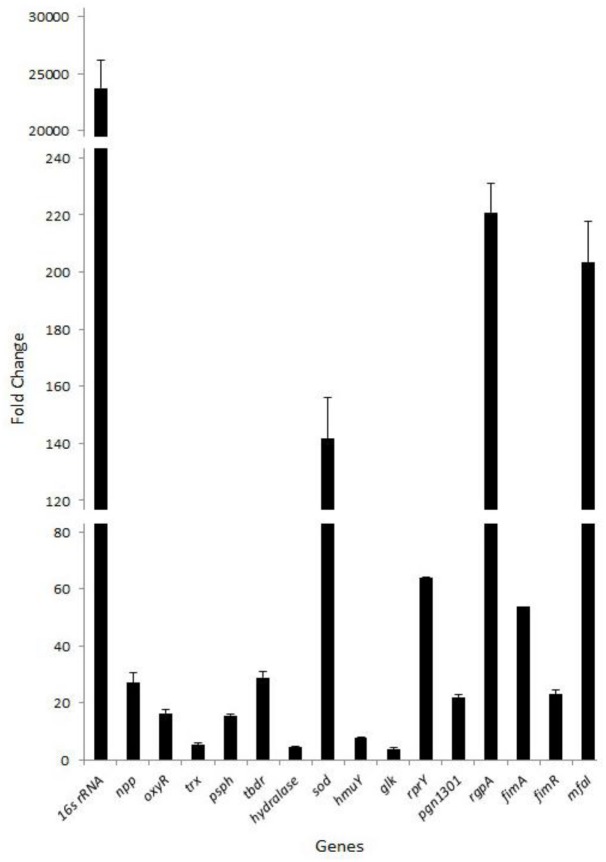
Detection of RNAs in *P*. *gingivalis* vesicles. RNA associated with vesicles from *P*. *gingivalis* 33277 was identified with a reverse transcription and qPCR analyses. Each bar represents the fold increase of DNA fragment levels detected after reverse transcription and qPCR analyses normalized by those detected in the vesicles without a reverse transcription. Values represent means ± SD of three replicates.

To determine if RNAs are also incorporated into *P*. *gingivalis* vesicles, RNAs were isolated from purified vesicles of *P*. *gingivalis* 33277 and were reverse-transcribed. qPCR was performed with the same set of primers used for DNA detection. RNA sequences from the open reading frames of 16 genes (S1 Table) were all recovered using RT-PCR (Fig 3). Not surprisingly, there was a very high rate of detection of 16S rRNA, followed by a number of the mRNAs of well-known virulence factors such as *mfa1*, *sod*, and *fimA*.

### DNA transfer through vesicles in *P*. *gingivalis*

**Fig 4:**
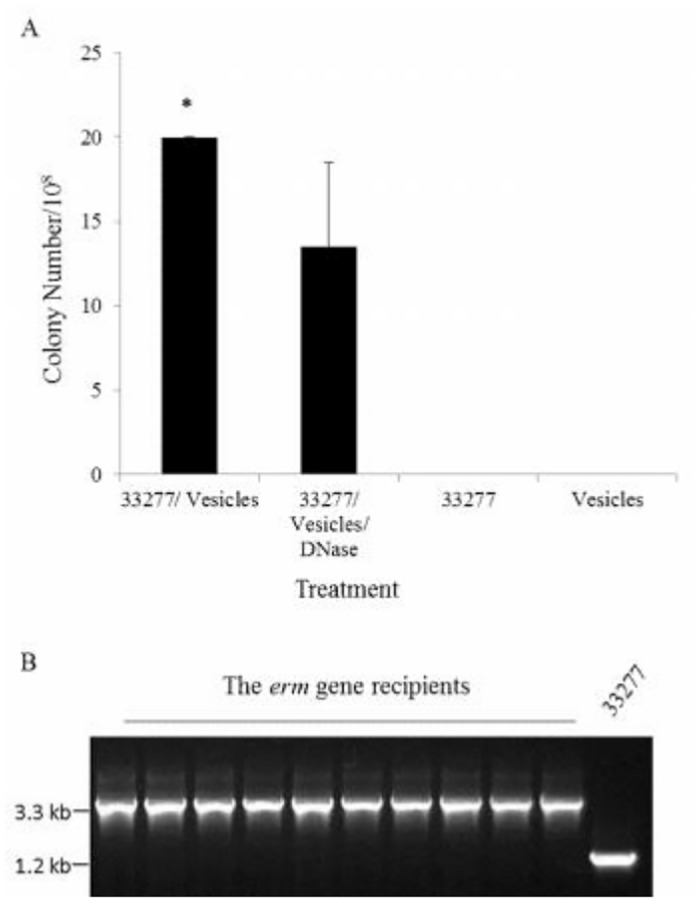
Horizontal gene transfer in *P*. *gingivalis* strains. Vesicles carrying an erythromycin resistant gene were derived from a *fimA* mutant and used as donors. *P*. *gingivalis* 33277 was the recipients. (A) The *P*. *gingivalis* 33277 receiving the *erm* gene was selected on TSB blood plates supplemented with erythromycin. Each bar represents the number of colonies detected on the plates, after *P*. *gingivalis* 33277 was incubated with vesicles without DNase pre-treatment, vesicles with DNase pre-treatment, and *P*. *gingivalis* 33277 without vesicles respectively, or vesicles alone without *P*. *gingivalis* 33277. An asterisk indicates a significant difference between colonies detected in *P*. *gingivalis* 33277 incubated with the vesicles without DNase treatment *vs*. those found in other groups (*n* = 3, *p* <0.05; *t* test). (B) The *erm* gene was detected by PCR with the *fimA* primers as a probe, and PCR products were visualized on agarose gels. The PCR product of the *fimA* gene is shown at 1.2 kb, and the PCR product of the chimeric gene (*fimA* + *erm*) is at 3.3 kb.

Vesicle-mediated horizontal gene transfer has been reported in some Gram-negative bacteria including *Acinetobacter baumannii* and *E*. *coli* [20,21]. To test if DNA detected in *P*. *gingivalis* 49417 (type III *fimA* strain) vesicles may be transferred to *P*. *gingivalis* strain 33277 (type I *fimA* strain), we isolated vesicles from a *fimA* mutant derived from *P*. *gingivalis* 49417. This mutant carries a 2.1-kb *ermF-ermAM* cassette encoding an erythromycin resistant gene incorporated into the *fimA* gene [22]. PCR analysis confirmed the presence of the *ermF-ermAM* cassette in *P*. *gingivalis* 49417 vesicles (data not shown), therefore, this antibiotic resistant gene was used as a marker to select any recipients. After incubating with purified vesicles from the *fimA* mutant for 24 h, *P*. *gingivalis* 33277 gained full resistance to erythromycin was detected with efficiency at 1.9 × 10^-7^. (Fig 4A). A lower efficiency was found when bacteria were incubated with the vesicles pretreated with DNase. Moreover, no erythromycin resistant colony was found on the plates inoculated with equivalent amount of *P*. *gingivalis* cells or vesicles alone. It should be pointed out that the efficiency is only based on the chance of *P*. *gingivalis* 33277 taking up the *erm* gene from the vesicles. Since multiple genes were packaged into the vesicles, genes other than the *erm* gene may also be transferred into *P*. *gingivalis* 33277. In addition, a difference was found when comparing the efficiencies of gene transfer when vesicles with or without DNase treatment were used as donors, suggesting that DNA fragments exist within vesicles and on the surface and both of them may be transferred into *P*. *gingivalis* cells.

After gene transfer into recipients, a gene must be integrated into the chromosomal DNA in order to persist within the cells. It is presumed that the *erm* gene in the vesicles of the *fimA* mutant is flanked with *fimA* sequences at both ends, and that homologous DNA recombination occurs between vesicle donor DNA and the chromosomal DNA of the recipient. To determine if the *erm* gene incorporated into the *fimA* gene of the recipient through an allelic-exchange, we analyzed the insertion sites of the *erm* gene using PCR with genomic DNA isolated from ten *P*. *gingivalis* recipients as templates, and primers corresponding to the promoter and termination regions of the *fimA* gene as probes. As shown in Fig 4B, the *erm* gene found in all recipients was incorporated into the *fimA* gene, since size of all PCR products is about 3.3 kb that includes full sizes of the *fimA* gene and the *erm* gene.

### Effect of *P*. *gingivalis* vesicles in *S*. *gordonii* biofilms

**Fig 5:**
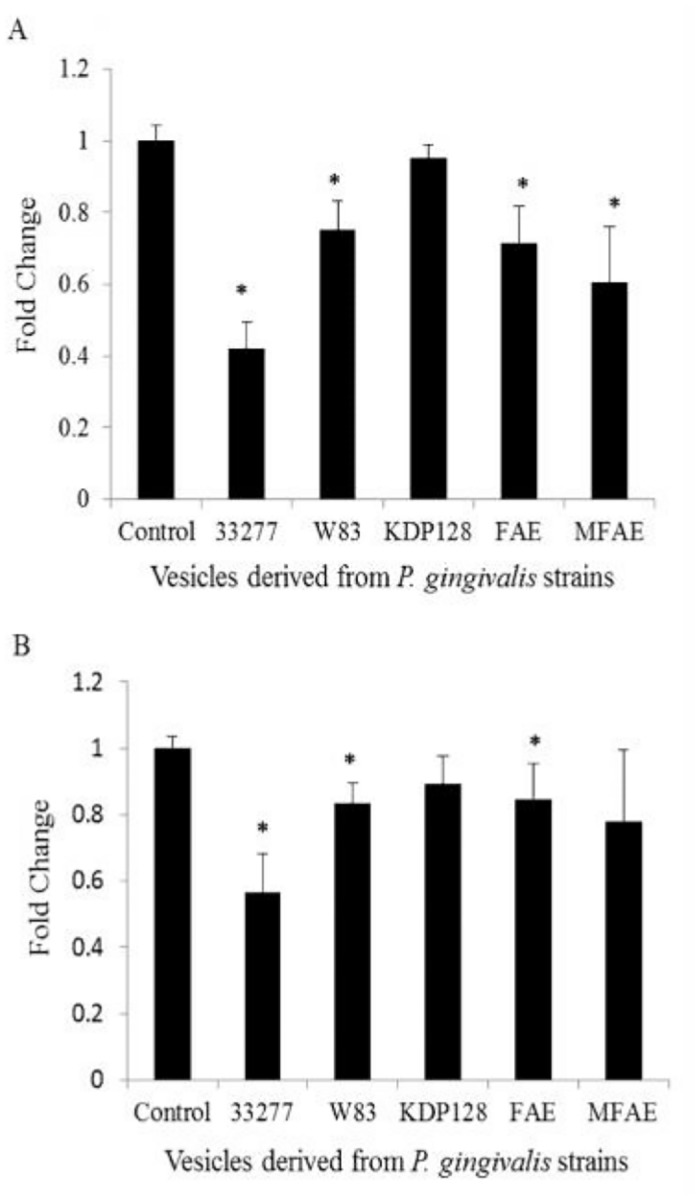
Inhibition of *S*. *gordonii* biofilm formation by *P*. *gingivalis* vesicles. *S*. *gordonii* DL1 was introduced to saliva-coated wells for establishment of *S*. *gordonii* biofilms. Vesicles derived from wild type strains 33277 or W83, the *fimA* mutant (FAE), the *mfa1* mutant (MFAE) or a gingipain-null mutant (KDP128), were either added into the wells at the beginning (A) or after *S*. *gordonii* biofilms were already formed (B). The *S*. *gordonii* biofilm remaining on the surfaces of the wells was determined, and the relative binding ability of each strain was represented and compared to that of wild type strain 33277 = 1 unit. The results presented are the averages of four independent experiments. Error bars represent SDs. An asterisk indicates there was a statistically significant difference in the biofilm formation between *S*. *gordonii* in the presence or absence of *P*. *gingivalis* vesicles (*P* <0.05; *t* test).

Co-aggregation between *P*. *gingivalis* and *S*. *gordonii* is well-known example of heterotypic biofilm formation. Previous studies revealed involvement of multiple sets of adhesins in the interaction of these two bacteria. The first set of adhesins includes *P*. *gingivalis* long fimbriae (FimA) and GAPDH present on the surface of streptococci [23]; the second set involves *P*. *gingivalis* short fimbriae (Mfa1) and streptococcal SspA/B (antigen I/II) adhesins [24,25]. As both FimA and Mfa1 are major proteins associated with *P*. *gingivalis* 33277 vesicles [14], we therefore examined the role of *P*. *gingivalis* vesicles in biofilm formation of *S*. *gordonii*. A biofilm formation assay was first performed by inoculating vesicles derived from *P*. *gingivalis* strains with *S*. *gordonii* DL1into the 96 well plates covered with human saliva for 48 hr. In the presence of vesicles from wild type 33277, there was a 60% decrease in the biofilm formation of DL1 compared to DL1 biofilm formation in the absence of vesicles. While, there was a 20–30% decrease in biofilm formation of DL1 formation in the presence of vesicles from wild type strain W83 (lacks fimA), the *fimA* mutant FAE, or the *mfa1* mutant MFAE (Fig 5A). However, there was no significant difference detected when comparing DL1 biofilm formation with or without the addition of vesicles from the gingipain-null mutant (*rgpA* ^*-*^, *rgpB* ^*-*^ and *kgp* ^*-*^) strain KDP128 [26].

The role of vesicles in biofilm disposal was then tested by the addition of vesicles after DL1 biofilms were formed. Similar results were obtained. Vesicles from wild type strain 33277 presented the highest ability to release and disperse DL1 cells from biofilms compared to W83, the *fimA* mutant. Addition of KDP128 or MFAE vesicles had no effect on DL1 biofilms (Fig 5B). These findings demonstrate that gingipains and fimbrial proteins are involved in inhibition of biofilm formation of *S*. *gordonii*. Presumably, fimbrial protein-mediated attachment of vesicles establishes an intimate relationship to *S*. *gordonii*, which in turn, facilitates the degradation actions of gingipains.

## Discussion

An intriguing feature of *P*. *gingivalis* is its ability to invade a variety of host cells. A comparison of the invasive capabilities of *P*. *gingivalis* cells and its vesicles revealed a significant higher invasive capability of vesicles. The invasive ability of *P*. *gingivalis* cells has been well documented [27,28]. *P*. *gingivalis* invasion is initiated by attachment to host cells, which is mediated by the interaction between the major fimbriae and surface receptors on host cells, for example primarily integrin α5β1 found on epithelial cells [29]. However, the mechanism (s) responsible for invasion of *P*. *gingivalis* vesicles is not well understood. There is existing evidence that a different mechanism(s) is likely involved in vesicle invasion, *per se*. Thus far fimbriae have not been observed on bacterial vesicles, but our previous studies showed that *P*. *gingivalis* vesicles consist of several well-known adhesins, such as fimbrial proteins and gingipains [14]. In addition, vesicles derived from the *fimA* mutant did not show any effect on their invasive capabilities compared to found in vesicles from the wild type strain, although the mutant still had a significantly decreased invasion capability compared to its parent cells [30]. Moreover, internalization of *P*. *gingivalis* cells has been reported to be involved in secretion of the serine phosphatase SerB, which can be found in host cells. SerB activates the actin-depolymerizing molecule cofilin by dephosphorylating the Ser 3 residue [31], resulting in a transient and localized disruption of actin structures that allow entry of the organism into the cytoplasm. Since SerB was not found in *P*. *gingivalis* vesicles [12,14], other unknown mechanism(s) may be responsible for the rearrangement of actin in this setting.

Horizontal gene transfer is known as one of the driving forces for the evolution of bacteria; it also represents an efficient step allowing bacteria to acquire new functions. Besides three well-known mechanisms of gene transfer (transformation, transduction, and conjugation), a fourth mechanism for horizontal gene transfer has been identified and involves bacterial vesicles. For example, vesicle-mediated gene transfer was found from the food borne pathogen *Escherichia coli* O157:H7 to *E*. *coli* JM109 and *Salmonella enterica* [32]. In a series of studies on horizontal gene transfer, Tribble *et al*. reported that DNA transfer occurs in *P*. *gingivalis* mainly through a transformation-like process, while a conjugation-like mechanism may only play a minor role [33–35]. Here, we demonstrated that vesicle-associated DNA was transferred between two strains of *P*. *gingivalis*, although the DNA transfer efficiency of vesicles pre-treated with DNase I was lower than that of vesicles in the absence of DNase I pre-treatment. Since native plasmids and bacteriophages have not found in *P*. *gingivalis*, it is not known how the chromosomal DNA is packaged into the vesicles. Berleman and Auer proposed three possible routes of incorporation of DNA into bacterial vesicles [36]: 1) cell-free vesicles passively take up extracellular DNA; 2) DNA is packaged into vesicles during the process of cell death; and 3) in some bacteria, DNA is taken up during bacterial replication through a budding process. However, there is no evidence that any of these mechanisms is used for DNA incorporation into *P*. *gingivalis* vesicles. Another important question is how DNA is released from vesicles into *P*. *gingivalis’* cytoplasm. Previous studies of *Pseudomonas aeruginosa* demonstrated that vesicles from this bacterium were fused to *E*. *coli*, which facilitated the attachment of these *E*. *coli* to *P*. *aeruginosa* parent cells [37,38]. Although there remains a lack of direct evidence for this process, we speculate that vesicles from the *fimA* mutant may merge with the outer membrane of *P*. *gingivalis* 33277 and release vesicular contents into the bacterial cells.

Finding DNA within *P*. *gingivalis* vesicles make them good candidates for induction of atherosclerosis. Atherosclerotic cardiovascular disease, a chronic inflammatory disease of the blood vessels, is one of the most common causes of morbidity and mortality world-wide [39]. Dysfunction of endothelial cells induced by hypertension, reactive oxygen species, or modified low-density lipoprotein cholesterol (LDL) express more adhesive molecules and promoted adhesion and infiltration by immune system cells [40]. Bacteria-mediated inflammation is also known to be, at least in part, responsible for the initiation of atherosclerosis. One of the best model pathogens in pathogenesis of atherosclerosis is *P*. *gingivalis* [41]. Involvement of *P*. *gingivalis* in atherosclerosis is supported by observations from epidemiological, clinical, immunological and molecular studies [42–46]. Some of the most convincing evidence of its role in atherosclerosis is the finding of *P*. *gingivalis* in cardiovascular samples including atheromantous plaques using PCR and in situ hybridization. A total of 21 studies from 2000 to 2012 found *P*. *gingivalis* DNA in 0–88.85% of cardiovascular samples, with a mean of 58% [41]. Studies that attempted to detect live *P*. *gingivalis* in atheromantous plaques were unsuccessful. However, a study by Kozarov *et al*. showed that both *P*. *gingivalis* and *A*. *actinomycetemcomitans* were detected within human coronary artery endothelial cells using immune-staining after cells were incubated with a homogenate of carotid atherosclerotic plaque sample from one patient [47]. Another report also discovered *P*. *gingivalis* colonies in one of seven homogenates of atherectomy specimens after a pre-incubation of the individual specimens with human monocytic cells, but not when directly incubated with homogenates [48]. It remains unknown how monocytic cells facilitated isolation of viable *P*. *gingivalis*. Based on our observations, including the efficient invasive activities and detection of vesicle-associated DNA, it is conceivable that vesicles are a major contributor to the development of atherosclerosis and that previous detection of DNA and proteins of *P*. *gingivalis* in atherosclerotic samples may most likely originate from *P*. *gingivalis* vesicles, although it cannot be ruled out that *P*. *gingivalis* cells may also disseminate to the arterial wall.

Dental plaque is a multi-species microbial biofilm. Interactions between/among different species are established by the specific recognition between adhesin and receptor. Most of the well-known adhesive molecules are found in *P*. *gingivalis* vesicles such as FimA and Mfa1. Therefore, vesicles, once released, likely act as representatives of *P*. *gingivalis* in order to communicate with other oral bacteria. We found that *P*. *gingivalis* vesicles functioned as an inhibitor and disperser of *S*. *gordonii* biofilms, which mainly involved gingipains, since a gingipain-null *P*. *gingivalis* mutant completely lost its inhibitory activity. *P*. *gingivalis* gingipains are multifunction proteins containing a proteolytic domain and adhesive domains, therefore, they may act as scissors and/or a glue in dental plaques. A previous *in vitro* study showed that lys-gingipain (Kgp) is involved in the degradation of synthetic peptides derived from the SspB protein of *S*. *gordonii* [49]. Beside an interaction with Mfa1 of *P*. *gingivalis*, *S*. *gordonii* SspB also play a role in the bacterial aggregation with *Candida albicans* through its interaction with the cell wall protein, Als3 [50]. It is likely that scissors and/or the glue function of *P*. *gingivalis* vesicles may create a favorable environment by eliminating competitors within the oral microbial community.

## Supporting Information

S1 Table Oligonucleotide primers used in this study. (PDF)
