# Supplementary material for: Functional Advantages of Porphyromonas gingivalis Vesicles
Source: PLoS One. 2015 Apr 21;10(4):e0123448. doi: 10.1371/journal.pone.0123448 (PMC4405273; doi:10.1371/journal.pone.0123448)
Supplement: S1 Table — (PDF) [file pone.0123448.s001.pdf]

S1 Table. Oligonucleotide primers used in this study

Gene	Primer name	Primer sequences (5'-3')
thioredoxin (trx)	Pg0275-81F	ttggcagggattttcggtgtcaga
	Pg0275-81R	gcccatcgtttgcgtcggtattc
superoxide dismutase (sod)	Pg1545-235F	aattccaccacggtaagcac
	Pg1545-235R	gagccgaattgtttgtcgat
glucose kinase (glk)	pg1737-131F	atgaatccgatccgccaccac
	Pg1737-131R	gcctcccatcccaaagcact
redox-sensitive transcriptional activator (oxyR)	pg0270-132F	tcgcaagccaagcaaatac
	pg0270-132R	gacacgaggcaggagatagg
hmuY	Pg1551-149F	gcacccacgagcttctttac
	Pg1551-149R	atacggaattgcccatgaag
phosphoserine phosphatase (psph)	Pg0653-F	agcgatcttgaagcctacca
	Pg0653-R	tctgtgatgcaggagattgc
arginine-specific cysteine proteinase (rgpA)	Pgn1970-160F	gtcaccgtctgcatcgatcg
	Pgn1970-160R	agtcggccagaaagtaacgc
DNA-binding response regulator (rprY)	Pgn1186-120F	gctcgttttctttggtcgtc
	Pgn1186-120R	gcggacgttcgaacaagtat
TonB-dependent receptor (tbdr)	Pg0707-147F	gccttgtacggttcttctgc
	Pg0707-147R	gacgatggaggcattgaagt
Pgn 1301	Pgn1301-F1	ccacacggatgataccctct
	Pgn1301-R1	gtactacgcgcagggaagaa
hydrolase	Pg1170-F	ttcgcacaacatcaaagagc
	Pg-1170-R	ccgatagacgacctgtcagca
nucleotide pyrophosphatase (npp)	Pgn0239F-127	tatgggcagagagtccaacc
	Pgn0239R-127	gtacatcgaggagccggata
fimR	fimR-124F	taaccacgggtagccatttc
	fimR-124R	gagtcgttctgctgctgttg
mfa1	mfa1 RT-F	cagatgggttgttgctca
	mfa1 RT-R	atggaaagtgctgctggtag
fimA	fimA1-392F	ctgtgtgtttatggcaaacttc
	fimA1-392R	aaccccgctccctgtattccga

fimA full length (fimA-FL)	FimA F 1200	ttgttgggacttgctgctc
	FimA R 1200	aaccccgctccctgtattc
16s rRNA	16s-rRNA F	tgggtttaaagggtgcgtag
	16s-rRNA R	caatcggagttcctcgtgat
